# Supplementary material for: Hollow Porous CoO@Reduced Graphene Oxide Self-Supporting Flexible Membrane for High Performance Lithium-Ion Storage
Source: Nanomaterials (Basel). 2023 Jun 30;13(13):1986. doi: 10.3390/nano13131986 (PMC10343264; doi:10.3390/nano13131986)
Supplement: Supplementary file 1 [file nanomaterials-13-01986-s001.zip › nanomaterials-2465002-supplementary.pdf]

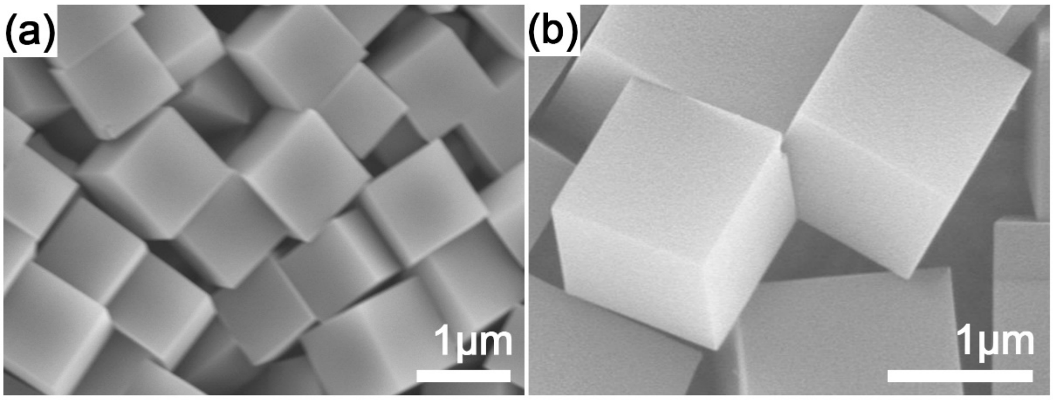


**Figure. S1** SEM images of Co-MOF nanocubes at different magnifications.

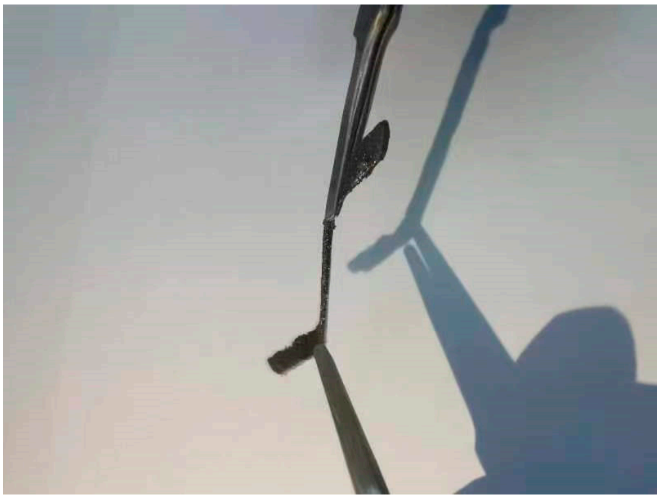

**Figure. S2** Digital images of CoO@rGO films ;

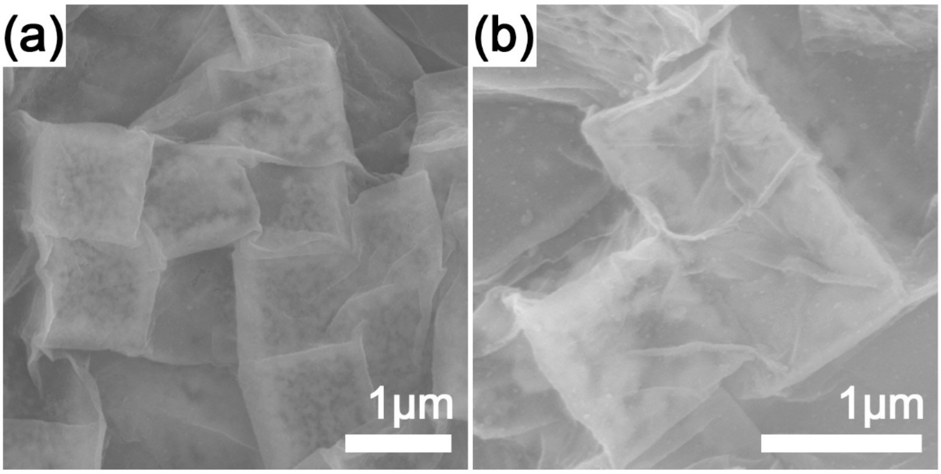


**Figure. S3** SEM images of hollow porous CoO@rGO flexible film

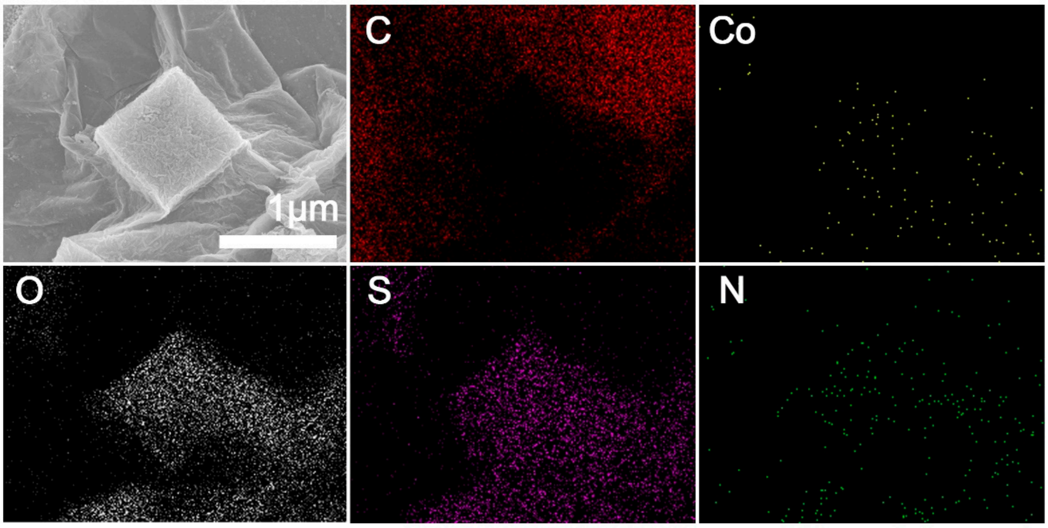


**Figure. S4** EDS analysis of the hollow porous CoO@rGO flexible film.

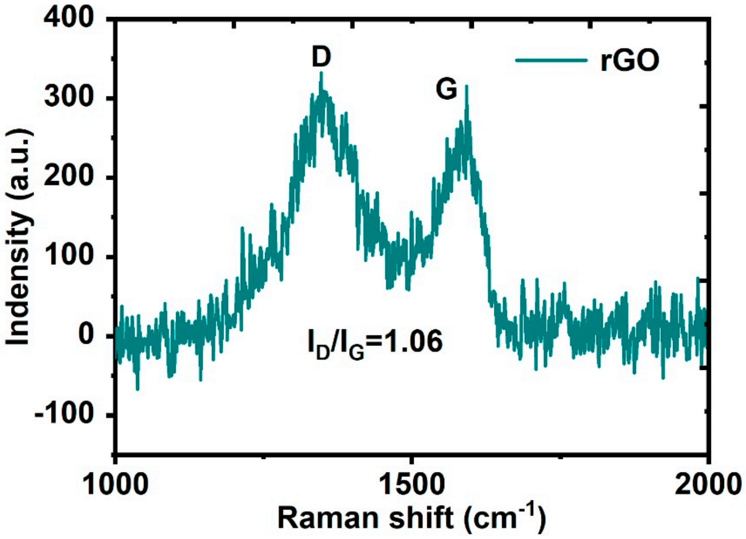


**Figure. S5** Raman spectra of rGo flexible film.

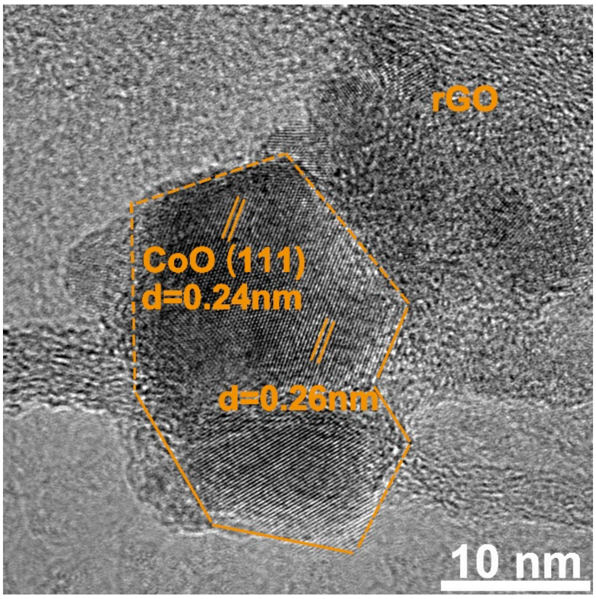


**Figure. S6** HRTEM image of Co-MOF@rGO

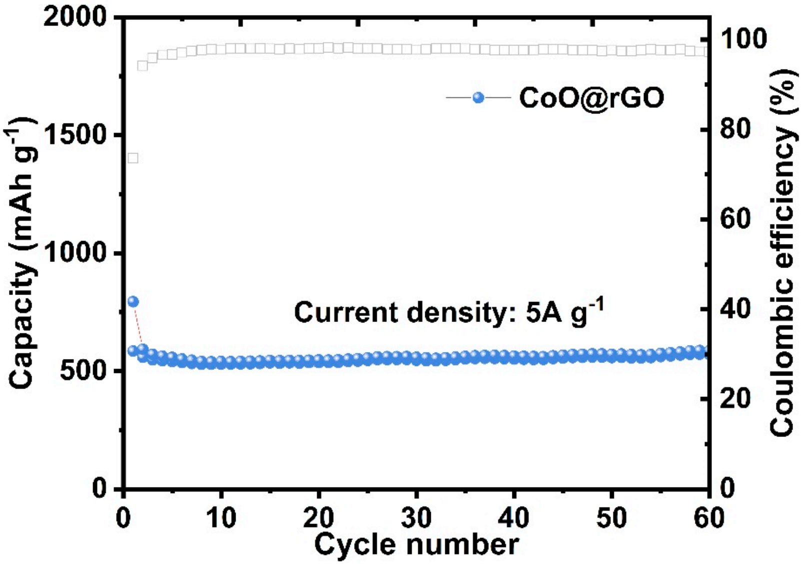


**Figure. S7** Cycling performance at 5 A g-1 of CoO@rGO flexible film for LIBs

Table S1 Comparison of electrochemical performances for this work.

| Electrode | Current density (mA $g^{-1}$) | specific capacity (mAh $g^{-1}$) | Cycle number (cycles) | Coulombic efficiency (%) |
|---|---|---|---|---|
| CoO | 1000 | 483 | 600 | 99.7 |
| rGO | 1000 | 155 | 600 | 99.7 |
| CoO@rGO | 1000 | 1103 | 600 | 99.1 |

| | | | | |
|---|---|---|---|---|
| CoO@rGO | 5000 | 586 | 60 | 97.2 |

Table S2 Test conditions and electrochemical performances of Co-based LIBs anodes.

| Electrode | Current density<br>(mA $g^{-1}$) | specific capacity<br>(mAh $g^{-1}$) | Cycle number<br>(cycles) | reference |
|---|---|---|---|---|
| CoO hollow spheres | 100 | 584 | 50 | 1 |
| CoO octahedral nanocages | 200 | 807 | 50 | 2 |
| Core-shellCo@CoO | 50 | 800 | 50 | 3 |
| $Co_3O_4$ Nps | 50 | 817 | 30 | 4 |
| $Co_3O_4$ nanocages | 50 | 970 | 30 | 5 |
| $Co_3O_4$@graphene | 200 | 941 | 60 | 6 |
| MWCNTs/$Co_3O_4$ | 1000 | 514 | 100 | 7 |
| Carbon-doped $Co_3O_4$ | 200 | 1121 | 100 | 8 |
| Hollow $Co_3O_4$/N,S-rGO | 1000 | 1590 | 60 | 9 |
| $Co_3O_4$/CuO nanowire | 1000 | 810 | 500 | 10 |
| CoO/$CoFe_2O_4$ | 1000 | 603 | 100 | 11 |
| $SiO_x$@C@CoO | 1000 | 714 | 750 | 12 |
| **This work** | **1000**<br>**5000** | **1103**<br>**586** | **600**<br>**60** | |

Reference：

1 Li, F.; Zou, Q.-Q.; Xia, Y.-Y. CoO-Loaded Graphitable Carbon Hollow Spheres as Anode Materials for Lithium-Ion Battery. Journal of Power Sources 2008, 177, 546–552, doi:10.1016/j.jpowsour.2007.10.097.

2 Guan, H.; Wang, X.; Li, H.; Zhi, C.; Zhai, T.; Bando, Y.; Golberg, D. CoO Octahedral Nanocages for High-Performance Lithium Ion Batteries. Chemical Communications 2012, 48, 4878–4880, doi:10.1039/C2CC30843F.

3 Zhang, L.; Hu, P.; Zhao, X.; Tian, R.; Zou, R.; Xia, D. Controllable Synthesis of Core–Shell Co@CoO Nanocomposites with a Superior Performance as an Anode Material for Lithium-Ion Batteries. Journal of Materials Chemistry 2011, 21, 18279–18283, doi:10.1039/C1JM12990B.

4 Wu, Z.-S.; Ren, W.; Wen, L.; Gao, L.; Zhao, J.; Chen, Z.; Zhou, G.; Li, F.; Cheng, H.-M. Graphene Anchored with Co3O4 Nanoparticles as Anode of Lithium Ion Batteries with Enhanced Reversible Capacity and Cyclic Performance. ACS Nano 2010, 4, 3187–3194, doi:10.1021/nn100740x.

5 Yan, N.; Hu, L.; Li, Y.; Wang, Y.; Zhong, H.; Hu, X.; Kong, X.; Chen, Q. Co3O4 Nanocages for High-Performance Anode Material in Lithium-Ion Batteries. J. Phys. Chem. C 2012, 116, 7227–7235, doi:10.1021/jp2126009.

.

6 Li, B.; Cao, H.; Shao, J.; Li, G.; Qu, M.; Yin, G. Co3O4@graphene Composites as Anode Materials for High-Performance Lithium Ion Batteries. Inorg. Chem. 2011, 50, 1628–1632, doi:10.1021/ic1023086.

.

7 Huang, G.; Zhang, F.; Du, X.; Qin, Y.; Yin, D.; Wang, L. Metal Organic Frameworks Route to in Situ Insertion of Multiwalled Carbon Nanotubes in Co3O4 Polyhedra as Anode Materials for Lithium-Ion Batteries. ACS Nano 2015, 9, 1592–1599, doi:10.1021/nn506252u.

8 Yan, C.; Chen, G.; Zhou, X.; Sun, J.; Lv, C. Template-Based Engineering of Carbon-Doped Co3O4 Hollow Nanofibers as Anode Materials for Lithium-Ion Batteries. Advanced Functional Materials 2016, 26, 1428–1436, doi:10.1002/adfm.201504695.

9 Zhu, J.; Tu, W.; Pan, H.; Zhang, H.; Liu, B.; Cheng, Y.; Deng, Z.; Zhang, H. Self-Templating Synthesis of Hollow Co3O4 Nanoparticles Embedded in N,S-Dual-Doped Reduced Graphene Oxide for Lithium Ion Batteries. ACS Nano 2020, 14, 5780–5787, doi:10.1021/acsnano.0c00712.

10 Wang, J.; Zhang, Q.; Li, X.; Xu, D.; Wang, Z.; Guo, H.; Zhang, K. Three-Dimensional Hierarchical Co3O4/CuO Nanowire Heterostructure Arrays on Nickel Foam for High-Performance Lithium Ion Batteries. Nano Energy 2014, 6, 19–26, doi:10.1016/j.nanoen.2014.02.012.

11 Li, M.; Yin, Y.-X.; Li, C.; Zhang, F.; Wan, L.-J.; Xu, S.; G. Evans, D. Well-Dispersed Bi-Component-Active CoO/CoFe 2 O 4 Nanocomposites with Tunable Performances as Anode Materials for Lithium-Ion Batteries. Chemical Communications 2012, 48, 410–412, doi:10.1039/C1CC15322F.

.

12 Wang, P.; Sun, Z.; Liu, H.; Gao, Z.-W.; Hu, J.; Yin, W.-J.; Ke, Q.; Lu Zhu, H. Strategic Synthesis of Sponge-like Structured SiO x @C@CoO Multifunctional Composites for High-Performance and Stable Lithium-Ion Batteries. Journal of Materials Chemistry A 2021, 9, 18440–18453, doi:10.1039/D1TA02880D
